# Determination of k-mer density in a DNA sequence and subsequent cluster formation algorithm based on the application of electronic filter

**DOI:** 10.1038/s41598-021-93154-3

**Published:** 2021-07-01

**Authors:** Bimal Kumar Sarkar, Ashish Ranjan Sharma, Manojit Bhattacharya, Garima Sharma, Sang-Soo Lee, Chiranjib Chakraborty

**Affiliations:** 1 Department of Physics, Adamas University, Kolkata, 700126 India; 2 Institute for Skeletal Aging and Orthopedic Surgery, Chuncheon Sacred Heart Hospital, Hallym University, College of Medicine, Chuncheon-si, Gangwon-do 24252 Republic of Korea; 3 Department of Zoology, Fakir Mohan University, Vyasa Vihar, Balasore, Odisha 756020 India; 4 Neuropsychopharmacology and Toxicology Program, College of Pharmacy, Kangwon National University, Chuncheon-si, Gangwon-do Republic of Korea; 5 Department of Biotechnology, School of Life Science and Biotechnology, Adamas University, Barasat-Barrackpore, Rd, Jagannathpur, Kolkata, West Bengal 700126 India

**Keywords:** Computational biology and bioinformatics, Evolution

## Abstract

We describe a novel algorithm for information recovery from DNA sequences by using a digital filter. This work proposes a three-part algorithm to decide the k-mer or q-gram word density. Employing a finite impulse response digital filter, one can calculate the sequence's k-mer or q-gram word density. Further principal component analysis is used on word density distribution to analyze the dissimilarity between sequences. A dissimilarity matrix is thus formed and shows the appearance of cluster formation. This cluster formation is constructed based on the alignment-free sequence method. Furthermore, the clusters are used to build phylogenetic relations. The cluster algorithm is in good agreement with alignment-based algorithms. The present algorithm is simple and requires less time for computation than other currently available algorithms. We tested the algorithm using beta hemoglobin coding sequences (HBB) of 10 different species and 18 primate mitochondria genome (mtDNA) sequences.

## Introduction

Over the last two decades, available DNA sequence data has grown exponentially. The understanding of the biological information and their implication with huge amount of DNA data has emphasized the crucial need for supportive sequencing methodologies. The development of cost-effective, efficient, and fast methods for sequence study is highly demanded. Substantial information for DNA sequence that put forward the arrangement of sequences variability indicates that the quantum of sequences that might have occurred throughout history is fewer in comparison to all possible sequences^1,2^. For example, considering 100 bp DNA sequence, one can construct as much as 4^100^ possible sequences. It demonstrates that only few of them thus exist in reality. The four letters A, C, G and T which denotes the four nucleotides, make the alphabetic sequence for coding genetic information. The genome sequences which consist of different gene can be readable by computer. Dealing with DNA sequence with computer becomes one of the prime domains in bioinformatics. It has vast application in data mining, comparative genomics, molecular phylogeny and genome annotation.

There are two types of methodology followed in sequence analysis-one is based on alignment and other is alignment free. In case of closely related sequence comparison, alignment-based methods are generally used. While dealing with divergent sequence it is better to use alignment free method^3–6^. On the other hand, alignment-based approach needs multiple or pairwise sequence alignments. Analyzing large datasets with an alignment-based method can surpass computational resources. Even sometimes, the combinatorics of genomic reorganisations makes the alignment of whole genomes quite difficult. A comparison of the alignment-based and alignment-free algorithm is given in Table 1.

**Table 1:** Comparison of the alignment-based and alignment-free algorithm (present study).

	Alignment based method	Alignment free method (Present Study)
Processing time	It needs vast CPU time which is proportional to N^K^. K is the number of sequences for alignment and N is the length of each sequence	It is based on successive two-dimensional dynamic programme for nucleotide density determination. It takes less CPU time which is proportional to N^2^
Handling in divergent sequence	Alignment-based approach works well for closely related sequences. In case of divergent sequences, a reliable alignment is difficult to be obtained	Alignment-based approach is workable for divergent sequences efficiently
Editing in sequence	This method requires editing in the sequence	This method does not require any editing in the sequence
Nature of algorithm	It requires dynamic programming, which is computationally expensive, to obtain alignment with optimal score	It relies on dynamic programmingby indexing word counts, without any optimization
Homology search	This method assumes the contiguity of homologous regions in the sequence	The assumption of homologous region contiguity is not required in alignment-free method

The alignment-free approach is independent in respect to the position of the strings. It is advantageous to compare sequences avoiding the biasness which arises from the order of strings within the sequences^7^. Two alignment-free methods, (a) graphical representation and (b) q-mer/word frequency estimation, are very popular for sequence similarity analysis. The visual inspection of data can be done through graphical representation. It facilitated to comparison of DNA sequences as well similarity analysis^8–10^. Different graphical representation has been reported to determine multi-dimensional mode for DNA characterization^11–18^ which are mostly two- and three-dimensional in nature. Li et al., reported 2D graphical representation of DNA sequence to constructed a coronavirus phylogeny^19^. Similarly, two-dimensional representation was described in dual nucleotides sequencing by Liu et al.^20^. Randic and Bai used triplet codons to obtained 2D graphical plot of protein sequences^21,22^. On the other hand three-dimensional graphical representation was used for different sequences. In this way RNA secondary structure was described using 3D graphical representation^23^. Cao et al*.* used dual nucleotide coding in three-dimensional representation^24^. Tri-nucleotide coding was also used in 3D graphical representations^25^. 4D/5D graphical representation was also reported by some workers to determining similarity index between two sequences^26–28^.

On the other hand, *q-mer*/word frequency estimation is based on the fact that the more alike two sequences are, higher the number of features that are common to two sequences. Blaisdell determined sequence comparisons based on frequency statistics It is very useful for the comparision of long sequences based on alignment-free statistic D2^29^. D_2_ analysis is based on finding correlation between the occurrences of all q-mers which appear in two sequences. But, D_2_ calculation is sensitive to noise arising from the sequence randomness. It results in low statistical power to determine relationship in the comparison of two sequences^30^. Sometimes word frequency measurement has low sensitivity when determining the statistical significance of a word’s frequency in the sequence^31–33^.

To reduce the computational complexity and find a more realistic method for the recovery of DNA sequence information, in this paper, we propose a digital signal processing (DSP) technique^34–36^. We impose a DSP-based finite impulse response (FIR) filter^37^ on a DNA sequence to calculate the k-mer or q-gram word density via sequence-free analysis. K-mer is highly important to understand the landscape of NGS read dataset^38^. The DSP technique is advantageous as it deals with the general structures of coding, and it is quite different from organism dependence. The four letter alphabet A, C, G, T are the basis to construct the genetic word of different lengths, viz., {AA, …, TT}, {AAA, …, TTT}. The first set of word is of length 2 and the latter set is of length 3. These are the examples of two letters and three letters word respectively. Generally, a *q*-nucleotide word is termed as *k-mer* or *q-gram*. As the set of DNA alphabet contains four letters, the quantity of all possible *q-grams* is *4*^*q*^. We use a dynamic window of width W to slide over the sequence to count the frequency (or occurrence) of each k-mer or q-gram. The window slides over one by one site. While existing on W number sites, digital filter technique is used to calculate the density of the word through the sequence. The distribution of word density along the sequence is taken as input for evaluation Principal Component Analysis (PCA), which helps finding the dissimilarity between the sequences. PCA projects the multi-dimensional data sets into low dimension without damaging the original data matrix's reliability^39,40^. To examine the validity of the method, we have taken two different groups, beta hemoglobin genes (HBB) and mitochondria genomes (mtDNA).

## Materials and methods

### Nucleotide Density

We have used *W*-size window which slides over the sequence in a base-by-base means between position *i* = 1 to *n* along the DNA strand. Window performs the counting of genetic word to find the word density in the sequence. The method relies upon the observations through sliding ‘counter’ of size *W* over the DNA sequence. A particular number of *q-grams,* herein called bins, are taken into consideration for the formation of the counter. The following definitions are helpful to calculate the word density distribution in a sequence.

#### Definition 1. q-gram of Sequence

Consider a counter of length q moves along a sequence segment ‘seq’ and it will count the signature of a q-gram. Thus, it will count a total number of $\left| {seq} \right| - \left( {q - 1} \right)$ q-grams over the sequence ‘seq’.

Since there are four basis of genetic alphabet, one can construct a total number of 4^*q*^ possible *q-grams* or *bins*. The bins are arranged in lexicographical order. If *i*^th^ bin is denoted by *b*_*i*_ in this order, the set of all possible bins are denoted as

$$ B_{q}  = \{ b_{1} ,b_{2} , \ldots b_{{4q}} \} $$

#### Example 1

*B*_1_ = {A, C, G, T}, consisting of 4 bins, represents the set of One-gram bins. For two-gram bins, there are 16 bins as represented by *B*_2_ = {AA, AC, AG, AT, CA, CC, CG, CT, GA, GC, GG, GT, TA, TC, TG, TT}.

#### Definition 2. Bin signature

Bin signature represents the presence or absence of a bin *b*_*j*_ $\left( {b_{j}  \in B_{q} ;j = 1,2,...,4^{q} } \right)$ at a position α in the sequence. It can be expressed as S_*j*_ , which is a mapping of bin *b*_j_ with is signature at a position α in the sequence. For a sequence segment ‘*seq*’, there are $\left| {seq} \right| - \left( {\left| {b_{j} } \right| - 1} \right)$ number of bits in S_*j*_.

#### Example 2

Consider a sequence, *seq* = “AACTCG”. The mono-gram (q = 1) bin signatures are S_A_ = [1 1 0 0 0 0] for the letter A, S_C_ = [0 0 1 0 0 0] for the letter C, S_G_ = [0 0 0 0 0 1] for the letter G, and S_T_ = [0 0 0 1 0 0] for the letter T. There are 4 mono-gram bin signatures. Similarly, the two-gram (q = 2) bin signatures are S_AA_ = [1 0 0 0 0], S_AC_ = [0 1 0 0 0], S_AG_ = [0 0 0 0 0], S_AT_ = [0 0 0 0 0], …, and S_TT_ = [0 0 0 0 0]. There are 16 two-gram bin signatures.

#### Definition 3. Filter

An input sequence *x*[*n*] undergoes a filter through weighted sliding window *b* to produce an output sequence *y*[*n*] by applying convolution summation, as follows:

1 $$ y\left[ n \right] = \sum\limits_{{i = 0}}^{k} {b_{i} x\left[ {n - i} \right]} $$

where b does not depend on *x*[*n*] and *y*[*n*], and *n* is the time-like index. *y*[*n*] is the response of the filter to the input signal *x*[*n*]. The finite impulse response (FIR) type filter is taken into consideration. The finite impulse response arises because the filter output is calculated as a weighted, finite term sum of the past and present.

#### Example 3

The weighted filter output of S_A_ with the window b = [0.2 0.1 0.3 0.4] is illustrated as follows:

S_A_ = [1 1 0 0 0 0].

$$y_{A} \left[ n \right] = \sum\limits_{0}^{3} {b_{k} S_{A} \left[ {n - k} \right]}$$ With b_0_ = 0.2, b_1_ = 0.1, b_2_ = 0.3, b_3_ = 0.4.

$$ y_{A} \left[ n \right] = b_{0} S_{A} \left[ n \right] + b_{1} S_{A} \left[ {n - 1} \right] + b_{2} S_{A} \left[ {n - 2} \right] + b_{3} S_{A} \left[ {n - 3} \right] $$

*y*_*A*_ = [0.2 0.3 0.4 0.7 0.4 0]; similarly the output for other nucleotides, viz., C, G, T, is computed as:

*y*_*C*_ = [0.2 0.0 0.2 0.1 0.5 0.5]; *y*_*G*_ = [0.0 0.0 0.0 0.0 0.0 0.2]; *y*_*T*_ = [0.0 0.0 0.0 0.2 0.1 0.3].

In the present work, we have considered uniformly distributed window of unit value for nucleotide density calculation. As elucidated, the output in the form of convolution summation denotes the nucleotide density distribution. The algorithms for bin structure, bin signature, and filter process are discussed as presented in different table (table S1, table S2, and table S3, correspondingly).

### Sequence analysis

Based on the density distribution of the DNA sequences, one can find the similarity/dissimilarity measure between two density distributions, *d*_*i*_ and *d*_*j*_ such that *d*_*k*_ = (*y*_*k1*_, *y*_*k2*_, …, *y*_*kn*_). A data matrix D is constructed by including all density distributions $\left[ {d_{1} ,{\kern 1pt} {\kern 1pt} d_{2} ,{\kern 1pt} {\kern 1pt} d_{3} ,...d_{m} } \right]^{\prime }$, where *m* is the total number of sequences. Thus, D becomes a m-by-n matrix. We wanted to locate the coordinates of the species in 2D and 3D representation. But the m-by-n D matrix cannot visualize the coordinates due to the higher dimensionality of the data set. In that case, reducing dimensionality to the utmost 3-dimension help visualization of the coordinates. In this scenario, we have used Principal Component Analysis (PCA) to reduce the multidimensional data sets to lesser dimensions without losing the consistency of the original data matrix. PCA helps estimating the scores between the density distributions. All the sequences under consideration are taken as a default query sequence in NCBI BLAST alignment as sense strands. In such a case, the nucleotide density distribution study is sufficient without emphasis on whether the strand is sense (positive) or antisense (negative).

**Figure 1:**
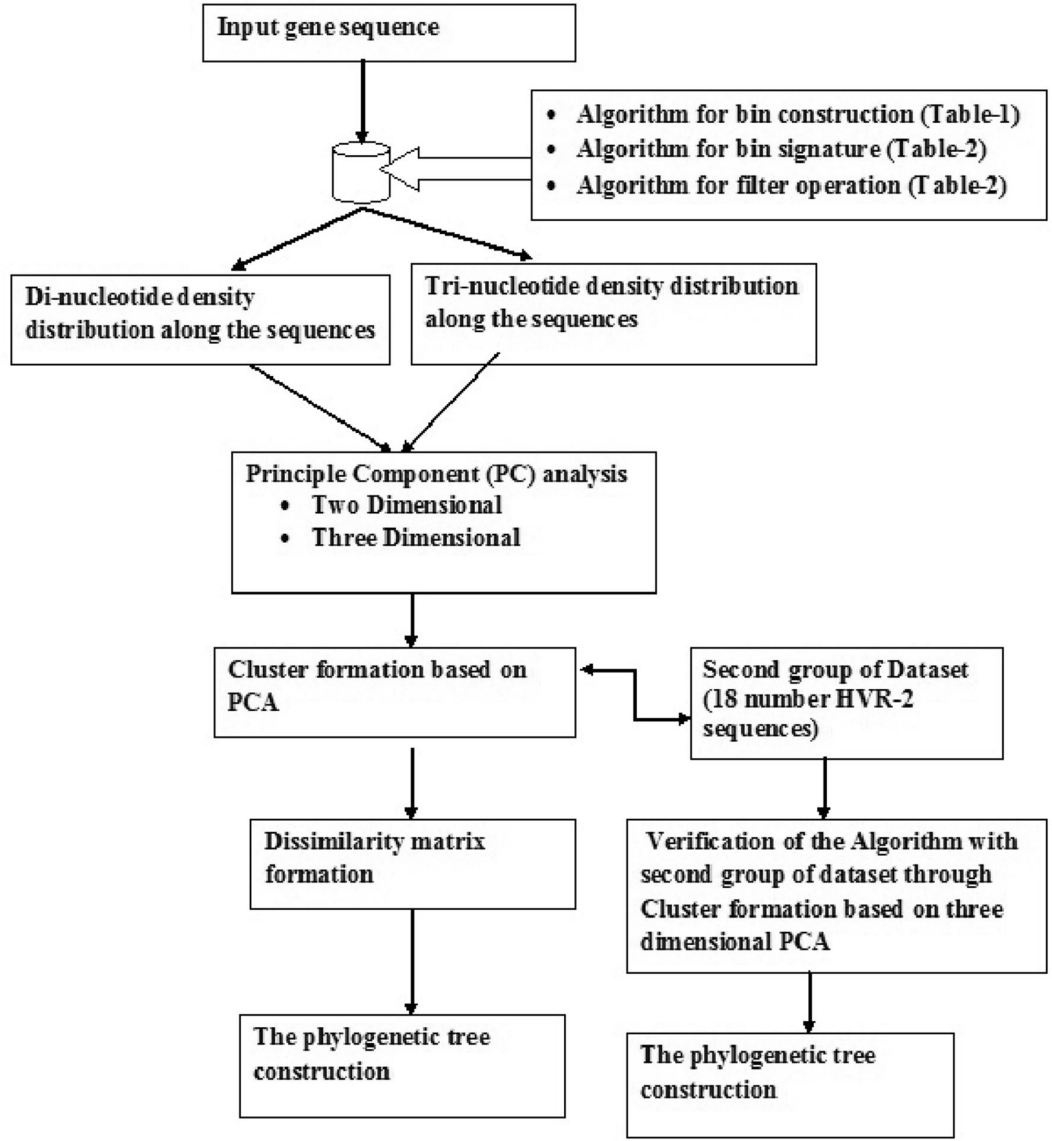
Execution flowchart of the algorithm.

The scores in the first three principal components are used to determine the dissimilarity between two sequences. Hence a score matrix, S, of m-by-3 order is constructed. Ordered pair of rows of the score matrix is taken for computation of the Euclidean distance between the pairs of sequences. Rows of S corresponding to sequences are the observations. On the other hand, the columns corresponding to the position index in the sequence are the variables. Since there are *m* number of observations, one can construct *m(m–1)/2* number of Euclidean distances, corresponding to pairs of observations in S. The Euclidean distances are organized in the order *(2, 1), (3, 1), …, (m, 1), (3, 2), …, (m, 2), …, (m, m–1)* and they are arranged in a row matrix of 1-by- *m(m–1)/2* order, which is further used for building dissimilarity matrix in clustering or multidimensional scaling. In our case, the low dimensional data in the first three components are taken for computation of the Euclidean distance between the pairs of sequences. Hence PCoA, K-medoid, or MDS are not required for further analysis^61^. A phylogenetic tree is constructed by employing Unweighted Pair Group Method with Arithmetic mean (UPGMA) on the PC scores as included in S matrix. The entire process is displayed as a flowchart in Fig. 1**.**

## Results and discussions

### Sequences

We have tested the algorithm with two sets of sequences- beta hemoglobin coding sequences (HBB) of 10 different species and 18 primate mitochondria genome (mtDNA) sequences. The detail of the sequences is given in Table S4 and S4 respectively. The sequences are obtained from NCBI genetic sequence database, which is publicly available, and it does not need any ethical approval. We have taken the Nucleotide database from NCBI. The nucleotide sequence is used to determine the nucleotide density distribution over the sequence. We considered sequences of beta hemoglobin coding genes (HBB) of 444 base-pair length from different organisms, such as primates, ungulates, rodents, and birds, to examine our algorithm. Similarly, the second set of sequence data of 337 base-pair length is taken from some primates' HVR-2 mitochondria genome. The sequences are of equal length. The study limits the comparison of sequences with different lengths. It can be overcome by normalizing sequence distribution. Moreover, in our case, a multiple sequence alignment algorithm is used to identify the aligned regions between the sequences.

#### FIR filtering

Employing FIR filter, the spatial distribution of nucleotide density is generated as *d*_*k*_ = (*y*_*k1*_, *y*_*k2*_, …, *y*_*kn*_), where *k* = 1 to 10 and n = 444. We calculated the density distribution for one-, two-, three-, … gram nucleotides for different organisms. Figure S1 displays the spatial variation of the nucleotide density along the HBB sequence for 10 organisms. The G nucleotide distribution demonstrates a rich density in base position between 70 and 100 for all organisms except *Gallus gallus.* The same distribution for the T nucleotide demonstrates a rich density of approximately 150 bp for most of the organisms except *Gallus gallus* and *Sus scrofa*. *Mus musculus* and *Rattus norvegicus* are enriched with A nucleotides in the region between 200 and 240 bp, but *Gallus gallus* shows enrichment of the A nucleotide in the region between 240 and 280 bp. *Gallus gallus* also shows enrichment of the C nucleotide in the region between 145 and 200 bp. This type of variation in nucleotide density profiles indicates the occurrence of evolution among organisms. In the subsequent discussion, we will elaborate on the variations through the formation of phylogenetic relations.

#### Di-nucleotide density distribution

We calculated 16 two-gram nucleotide densities along HBB sequences for 10 organisms. Figure S2 presents the AA and AC density distributions along the sequence for all organisms. *Mus musculus* and *Rattus norvegicus* show a high density of AA in the region between 195 and 245 bp. *Gallus gallus* shows enrichment of the AA di-nucleotide in the region between 245 and 285 bp. The density distribution of AC for *Homo sapiens* and *Gallus gallus* both show enrichment of approximately 275 bp.

#### Tri-nucleotide density distribution

We determined 64 three-gram nucleotide density profiles. Figure S3 shows two three-gram nucleotide distributions along the HBB sequences for 10 organisms. In the ACT distribution, *Macaca mulatta* and *Pan troglodytes* have high density regions at the very beginning of the sequence. *Homo sapiens* gas a high density at approximately 280 bp. In the ACT distribution, *Sus scrofa* has a high density at 225 bp and *Gallus gallus* has the same at approximately 290 bp, but *Callithrix jacchus, Equus caballus, Homo sapiens*, and *Sus scrofa* have high densities at approximately 325 bp.

### Principal component analyses (PCA)

#### Two component analysis

**Figure 2:**
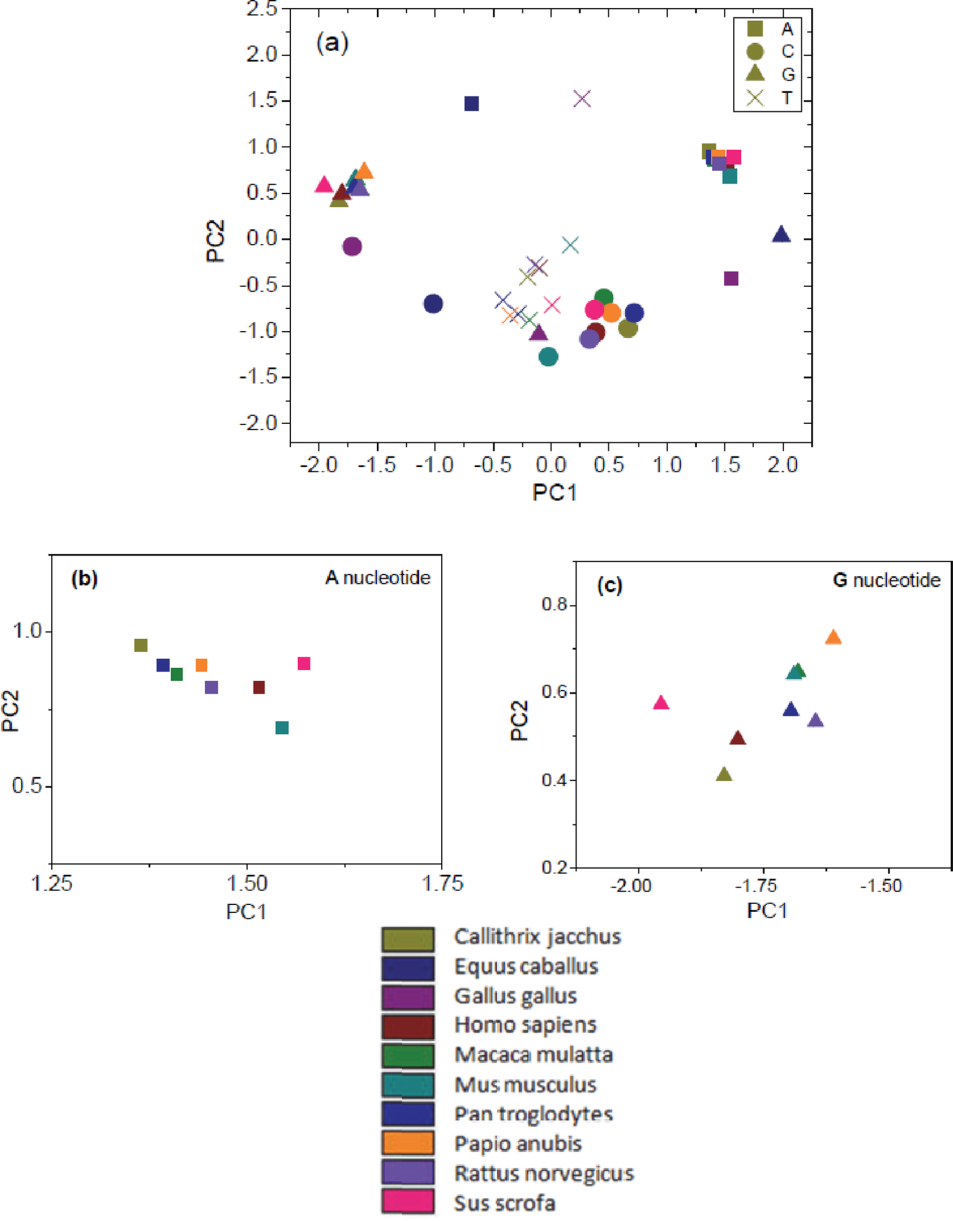
PC plot of for single nucleotide of 10 organisms. Circle: **A** nucleotide, Circle: **C** nucleotide, Triangle: **G** nucleotide, **X: T** nucleotide; Colour code represents different species as indicatedin the legend. Individual PC value for A and G nucleotide are shown in in panel (**b**) and (**c**) respectively.

Principal Component Analyses (PCA) is a vital technique frequently used in multivariate analysis to compare patterns by extracting information from a higher dimension to a lower dimension^41–43^. It is used in geometric morphometric shape analysis and consequently to determine trait-based phylogenic trees. PCA on phenotypic variance are found in the literature^44–46^. In our case, genome-wide patterns are taken as input to PCA which has been employed on the nucleotide density distribution along the sequences. PCA converts the information in multidimensional data sets into principal components (PC), which reduces the multidimensional data sets to a lesser dimension without losing the consistency of the original data matrix. Instead of considering all PCs, very few of the principal components are used to capture most of the original dataset variation. The data can then be represented in a 2D or 3D scatter plot, taking two or three principal component axes, respectively. This PCA plot helps visualization of groups of observations in the original dataset. First Principal Component (PC1) includes the most variation, PC2 represents the second most variation, and so on. In this way, the first two or three PCs are sufficient to capture most of the variation. We have considered a data set of m-by-n matrix for PCA, where m is the number of sequences and n is the length of each sequence. Each of m rows is projected on n number principal component basis. A scatter plot of the first two PC values dealing with HBB nucleotides of 10 organisms is displayed in Fig. 2. It was found that the majority of the variance is populated in the first three principal components. An average of 51.42% of the total variance is present in the first component. An average of 26.69% of the total variance is present in the second component, and an average of 13.89% of the total variance is present in the third component. Supplementary Table S7 exhibits the computed eigenvectors of the dataset along with the variance. The plot of two component PC values demonstrates the formation of cluster among the species. For example, the organisms *Homo sapiens, Rattus norvegicus, Pan troglodytes, Papio anubis, Sus scrofa, Mus musculus,* and *Callithrix jacchus* compose a cluster in PC values for the A nucleotide. The three other organisms, namely, *Equus caballus, Macaca mulatta,* and *Gallus gallus*, are isolated clusters. However, this type of cluster formation is not in agreement with the phylogenetic tree of HBB sequences, as reported by L. Yang et al*.*^47^. They showed the formation of four distinct clusters. Their clusters are (1) *Equus caballus, Sus scrofa*; (2) *Macaca mulatta, Papio anubis, Pan troglodytes, Callithrix jacchus, Homo sapiens*; (3) *Mus musculus, Rattus norvegicus*; and (4) *Gallus gallus*. After noticing this disagreement, we considered all four nucleotide density distributions simultaneously, in the same data matrix, and then performed three components PCA on the entire data matrix. We have included the script of the MATLAB code in the supplementary section (Script S1).

#### Three component analysis

**Figure 3:**
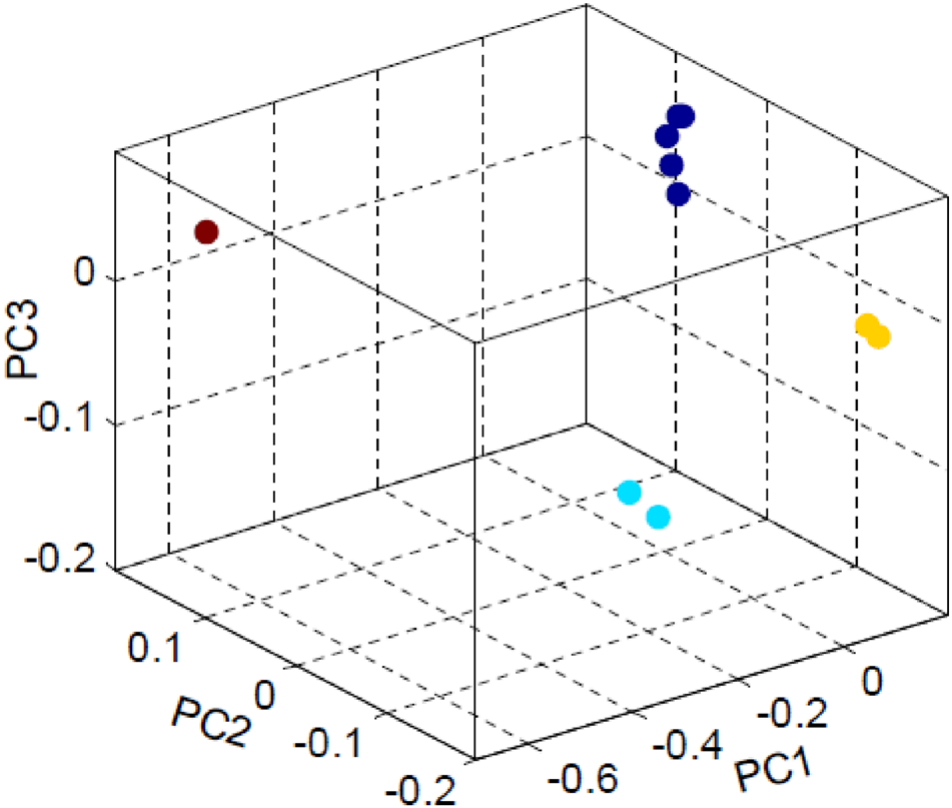
Scatter plot of three PC values. Circle (aqua color): Cluster 1 consists of *Equus caballus, Sus scrofa*; Circle (indigo color): Cluster 2 consists of *Homo sapiens, Callithrix jacchus, Pan troglodytes, Macaca mulatta, Papio anubis*; Circle (yellow color): Cluster 3 consists of *Mus musculus, Rattus norvegicus*; Circle (maroon color): Cluster 4 consists of *Gallus gallus*.

As mentioned earlier, a data set of the m-by-4n matrix is considered for 3D principal component analysis (3D-PCA). One can notice that instead of *n* columns, the data set contains 4n columns. It is due to the inclusion of four nucleotide density distribution in a single row of each sequence. Out of 4n columns, we have considered three columns, as three PCs contain most of the variation. Other columns (4n-3) are discarded as their contribution is less in the information acquirement. Finally, a data set of m-by-3 matrix is taken for 3D principal component plot as shown in Fig. 3. The PCA with simultaneous nucleotide density distributions is in good agreement with the phylogeny reported elsewhere^47^. Figure 3 clearly shows the formation of four clusters. Cluster 1 is composed of *Equus caballus* and *Sus scrofa*. Five organisms, *Homo sapiens, Callithrix jacchus, Pan troglodytes, Macaca mulatta,* and *Papio Anubis*, are included in cluster 2. Two organisms, *Mus musculus* and *Rattus norvegicus,* are grouped into cluster 3. Cluster 4, comprising only *Gallus gallus,* is far from the other clusters.

### Dissimilarity matrix calculation

**Figure 4:**
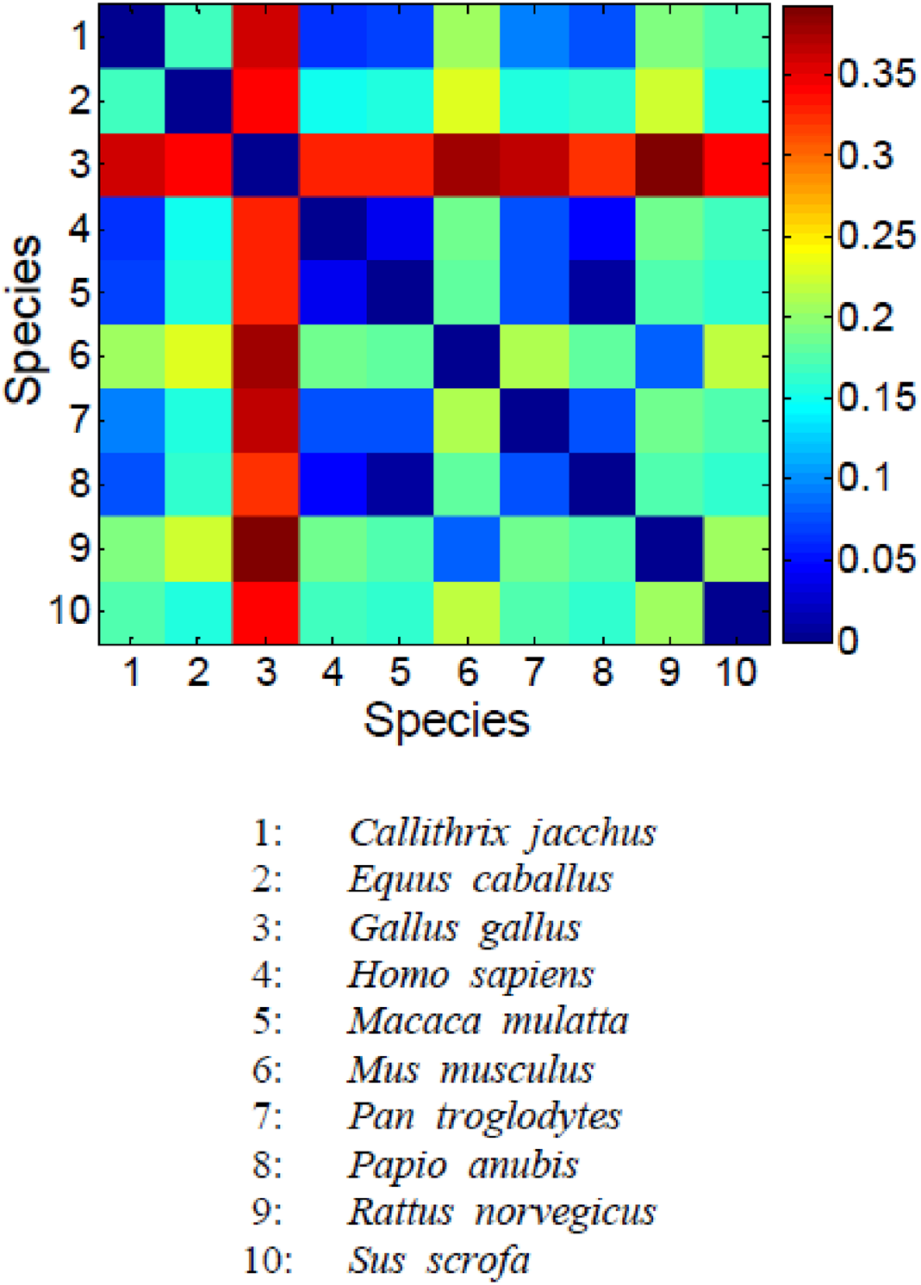
The dissimilarity matrix for beta HBB sequences derived from 10 organisms. The diagonal shows zero value, because diagonal element represents dissimilarity between an organism and organism itself.

The dissimilarity value (DV) between any two organisms was estimated based on the first 3 principle components (> 90% of the total variability) by computing the Euclidean distance between organisms. The obtained DV is used to find the dissimilarity between two sequences, as presented in table S5**.** Additionally, the obtained dissimilarity matrix is shown in Fig. 4. We observe that the DV of *Homo sapiens* from *Callithrix jacchus*, *Macaca mulatta*, *Pan troglodytes* and *Papio anubis* are, respectively, 0.063, 0.041, 0.077 and 0.049. On the other hand, the DV of *Homo sapiens* from the other species is greater than 0.15. The hemoglobin coding sequences of the organisms *Homo sapiens*, *Callithrix jacchus*, *Macaca mulatta*, *Pan troglodytes* and *Papio anubis* are very close, which is commensurate with the fact that they are all primates. The five primates constitute cluster 2, as mentioned in the PC scatter plot. The dissimilarity matrix for HBB sequences (Fig. 4) reveals dissimilarity between *Equus caballus* and *Homo sapiens* (DV = 0.151), which demonstrates two distant neighbor sequences. *Equus caballus* belongs to ungulates, which are different from primates. The organism *Mus musculus,* existing in the rodent species, maintains a DV of 0.189 from *Homo sapiens*. We also notice that *Gallus gallus* creates a very large DV with all of the organisms, with the smallest DV value being 0.324, which is quite obvious that *Gallus gallus* is a bird species. We have studied similarity based on Principal Component Analysis. PCA is not scale invariant. As a result, similarity rates are indicative.

### Phylogenetic tree

**Figure 5:**
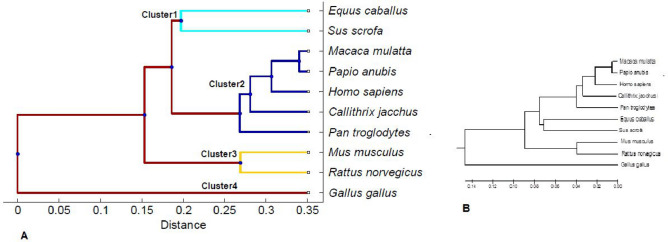
The phylogenetic tree of the hemoglobin coding sequences. (**A**) Phylogenetic tree is constructed with our algorithm (Alignment Free method). The similar sequences are grouped into cluster. Clusters are shown with different colours. (**B**) Phylogenetic tree using clustalW (Alignment based method).

Taking HBB sequences as a basis, we get the phylogenetic tree of the organisms with the Unweighted Pair Group Method's help with the Arithmetic mean (UPGMA) method as applied on the dissimilarity matrix. The phylogenic tree, as found from our algorithm, is displayed in panel A of Fig. 5. The same phylogenetic relationship is obtained by applying the CLUSTALW alignment-based method. It is indicated in panel B of Fig. 5. The two phylogenic tree as constructed based on two methods are in good agreement. Both the figures show the same nature of cluster formation. Cluster 1 includes two Ungulates accessions (*Equus caballus* and *Sus scrofa)*. Two members of cluster 1 are at a genetic distance (GD) of 0.307 from each other, which is relatively large in comparison to members in other clusters. This large GD between *Equus caballus* and *Sus scrofa* is shown to be relevant to the morphological criteria of different sides, namely, headgear, dentition, and foot structure^48,49^. They are mainly segregated on the basis of their foot morphology—whether they are even-toed or odd-toed hoofed mammals. *Equus caballus* is an odd-toed ungulate, and *Sus scrofa* is an even-toed ungulate. Cluster 2 contains five primates, namely, *Homo sapiens*, *Callithrix jacchus*, *Macaca mulatta*, *Pan troglodytes* and *Papio anubis*. They are close to one another in the tree, which means that primates maintain less GD in comparison to the other clusters. For example, the *Homo sapiens* and *Pan troglodytes* HBB sequences are almost identical, with a likeness of approximately 98.8% of their genome^50^. Phylogenic studies have established the relationships, presenting the common chimpanzee (*Pan troglodytes*) and bonobo (*Papio anubis*) as our neighboring evolutionary relatives^51^. However, some major interspecies evolutionary changes are not reflected in the short GD relationship^52^. It is found that *Gallus gallus* is genetically far away from the other organisms, with a GD as high as 0.7, which is in support of the fact that *Gallus gallus* is only non-mammalian species among the 10 species in the present study.

### Validation of the algorithm with another set of data

To further demonstrate the applicability of our method, we give emphasis to the origin of the human species. After the discovery of fossilized Neanderthal skeletons in Europe, many questions arose regarding the origin of human beings, from which the subject of our relation to Neanderthal is foremost. We apply the present sequencing method to study the mitochondrial genomes of primate origins. We choose mtDNA because of its rapid mutation rate^53^ and because it is worthwhile to elucidate the evolutionary relationships among species based on mtDNA sequence analysis. We consider the displacement loop (D-loop), which is a non-coding section of mtDNA. It comprises two regions, viz., hyper variable region 1 (HVR-1) and hyper variable region 2 (HVR-2), which represent variations between species. We have taken sequences from HVR-2. The associated mitochondrion genome sequences are downloaded from the GenBank database (table S6).

**Figure 6:**
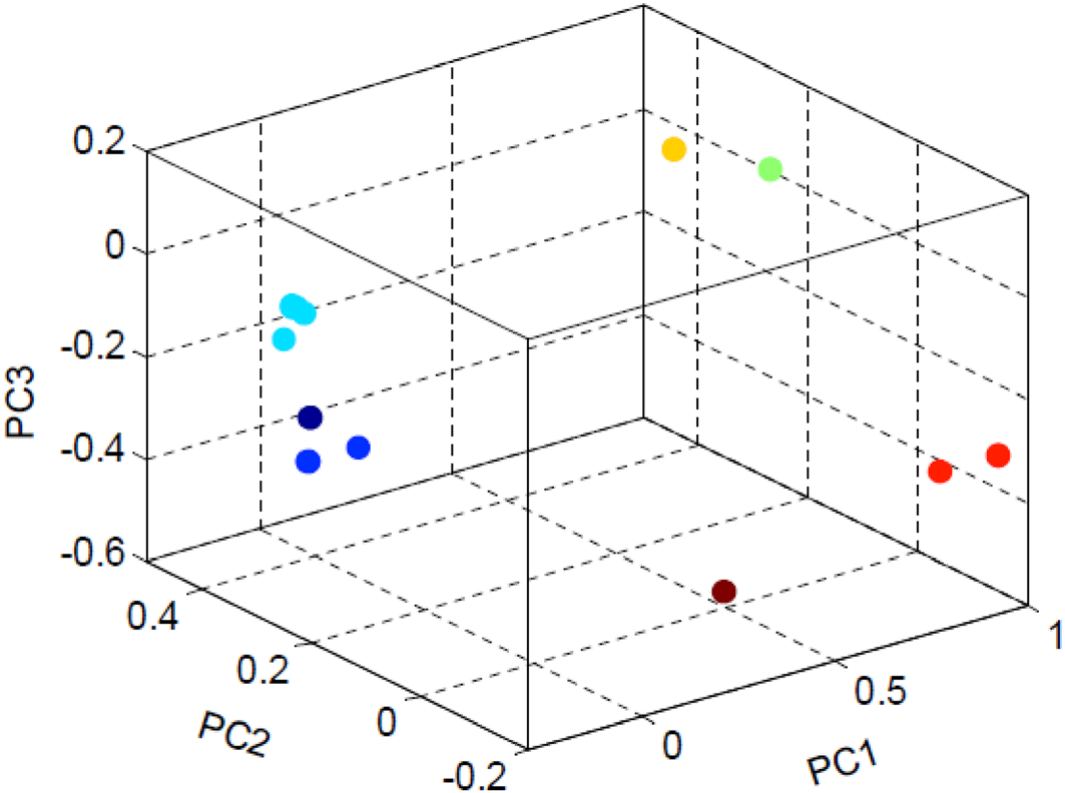
Scatter plot of the first three PC values. Circle (aqua color): Cluster 1 consists of Homo sapiens (*Berber*, *Chinese*, *Georgean, Yoruba*, and *Neanderthal)*; Circle (blue color): Cluster 2 consists of chimpanzees *(Pan_troglodytes_1, Pan_troglodytes_2, Pan_troglodytes_3);* Circle (indigo color): Cluster 3 consists of pygmy chimpanzees *(Pan_paniscus_1, Pan_paniscus_2)*; Circle (red color): Cluster 4 consists of gorillas *(Gorilla_gorilla_1, Gorilla_gorilla_2, Gorilla_gorilla_gorilla)*; Circle (yellow color): Cluster 5 consists of Sumatran orangutan (*Pongo_pygmaeus_abelii_1*, *Pongo_pygmaeus_abelii_1*); Circle (lime color): Cluster 6 consists of Bornean orangutan (*Pongo_pygmaeus_1, Pongo_pygmaeus_2*); Circle (maroon color): Cluster 7 consists of Gibbon *(Hylobates lar)*. Note that some data points are coincident due to close PC values of the organisms in the same cluster.

We examined the HVR-2 in the D-loop region of 18 species of primates, including four humans (*Berber*, *Chinese*, *Georgean,* and *Yoruba*), *Neanderthal,* three common chimpanzees, two pygmy chimpanzees, three gorillas, two Sumatran orangutans, two Bornean orangutans, and a gibbon, in an attempt to estimate the divergence of mtDNAs and to determine the relationship between them. We calculated the four nucleotide density distribution matrix followed by performing principal component analysis. The first three principal components for 18 primates are shown in Fig. 6. We observed that seven clusters were formed. Cluster 1 comprises five Homo sapiens (*Berber*, *Chinese*, *Georgean, Yoruba*, and *Neanderthal)*. Three chimpanzees *(Pan_troglodytes_1, Pan_troglodytes_2, Pan_troglodytes_3)* are included in cluster 2. Two pygmy chimpanzees *(Pan_paniscus_1, Pan_paniscus_2)* are grouped into cluster 3. Cluster 4 is built with three gorillas *(Gorilla_gorilla_1, Gorilla_gorilla_2, Gorilla_gorilla_gorilla)*. Sumatran orangutans (*Pongo_pygmaeus_abelii_1*, *Pongo_pygmaeus_abelii_1*) appear in cluster 5. Cluster 6 contains Bornean orangutans (*Pongo_pygmaeus_1, Pongo_pygmaeus_2*). Cluster 7 consists only of the gibbon *(Hylobates_lar)*.

**Figure 7:**
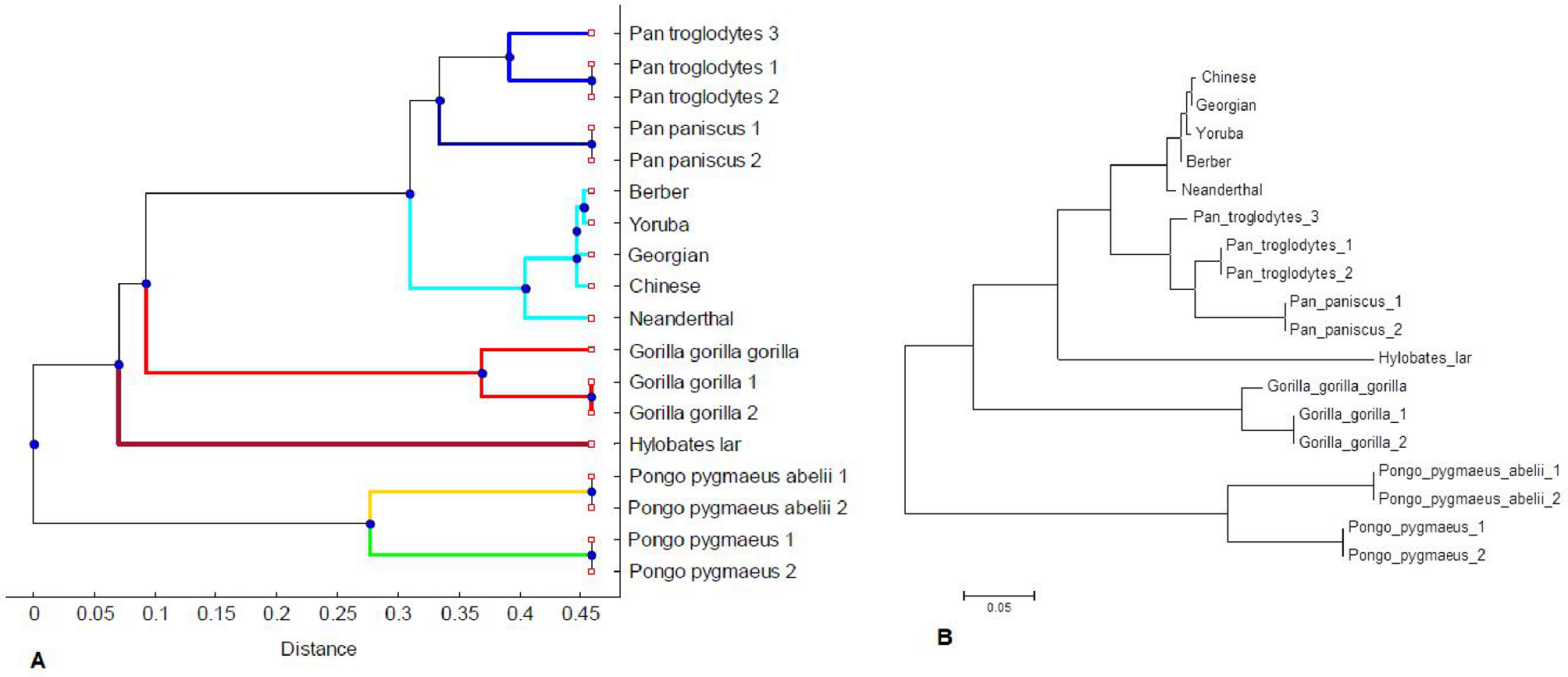
The mtDNA phylogenetic tree using 18 primate species sequences(test sequences). (**A**) Phylogenetic tree is constructed with our algorithm (Alignment Free method). The similar sequences are grouped into cluster. Clusters are shown with different colours. (**B**) Phylogenetic tree using clustalW (Alignment based method).

We have constructed a dissimilarity matrix of these sequences from the first three principal components, and the phylogenetic tree among the 18 organisms is obtained by using UPGMA algorithm. The phylogenic tree, as determined from the present algorithm, is shown in panel A of Fig. 7. It is also constructed by CLUSTALW alignment-based method. The latter tree is displayed in the panel B of Fig. 7. The two phylogenic trees as built by two methods show a good agreement. Both trees show the same nature of cluster formation. Our present phylogenetic analysis maintains consistency with the analysis reported by Cristianini and Hahn^54^. The organisms *Berber*, *Chinese*, *Georgean,* and *Yoruba* are very close in cluster 1. Neanderthals is shown to be connected to a common ancestor of Neanderthals and modern humans. However, there is controversy regarding the relationship between Neanderthals and modern humans^55^. Krings et al*.* described Neanderthal mtDNA to be far outside the phylogenetic tree connecting the mtDNAs of contemporary humans^56^. On the other hand, Wolpoff reported a close relation between Neanderthal and Europeans^57^. However, in our case, the mtDNA HVR-2 phylogenetic tree shows that Neanderthals are more closely related to modern humans than to any of the other extant Great Apes (chimpanzees, gorillas, orangutans) or Lesser Apes (gibbons). The GD between Neanderthals and the human line is 0.096687, whereas the GD between Neanderthals and chimpanzees is 0.29986. Gorillas and orangutans have GDs of 0.73339 and 0.91766, respectively, from Neanderthals. Gibbons are positioned at a GD of 0.77814 from Neanderthals. The common chimpanzee and pygmy chimpanzee have a GD of 0.2506 between them. The Sumatran orangutan has a GD of 0.36534 from Bornean orangutans. Lesser Apes are distinct from Great Apes; for instance, the gibbon is far from chimpanzees (GD = 0.77814).

The present clustering algorithm has several advantages over alignment-based methods. It does not require any pre-editing of the sequences. Alignment-based similarity findings may result in some type of incorrect relationship in the case of divergent sequences^58^. Comparisons of the existing alignment-based algorithms are provided in table S7**.** However Jemes et al. has developed a clustering for DNA sequences using clustering algorithm^59^. The present algorithm also helpful for genetic grouping of different species subtype using DNA sequence^60^.

## Conclusion

We propose a novel algorithm for the determination of the occurrence of *k-mer* or *q-gram* words in a DNA sequence for information recovery in sequence dynamics. The present algorithm counts the prevalence of each *k-mer* or *q-gram* occurrence by using dynamic window which slides over the sequence. Using digital signal processing (DSP) technique and based on the operation of a FIR type filter, we have calculated the density distribution which is further used for PCA. The present method is of sequence alignment-free and does not require graphical representation. The cluster algorithm based on the FIR operation validates its applicability. Application of the FIR algorithm demonstrates its applicability to two data sets with evolutionary importance, beta hemoglobin coding sequences (HBB) and HVR-2 mtDNA sequences. Our algorithm can be useful for various evolutionary analyses and will be very helpful for future biological communities that are working in the area of molecular phylogenetics and also helpful for genetic grouping of different subtype of a species using DNA sequence.

## Supplementary Information

Supplementary Information 1.

Supplementary Information 2.

Supplementary Information 3.

Supplementary Information 4.

Supplementary Information 5.

Supplementary Information 6.
