# Supplementary figures and images for: Determination of k-mer density in a DNA sequence and subsequent cluster formation algorithm based on the application of electronic filter
Source: Sci Rep. 2021 Jul 1;11:13701. doi: 10.1038/s41598-021-93154-3 (PMC8249421; doi:10.1038/s41598-021-93154-3)

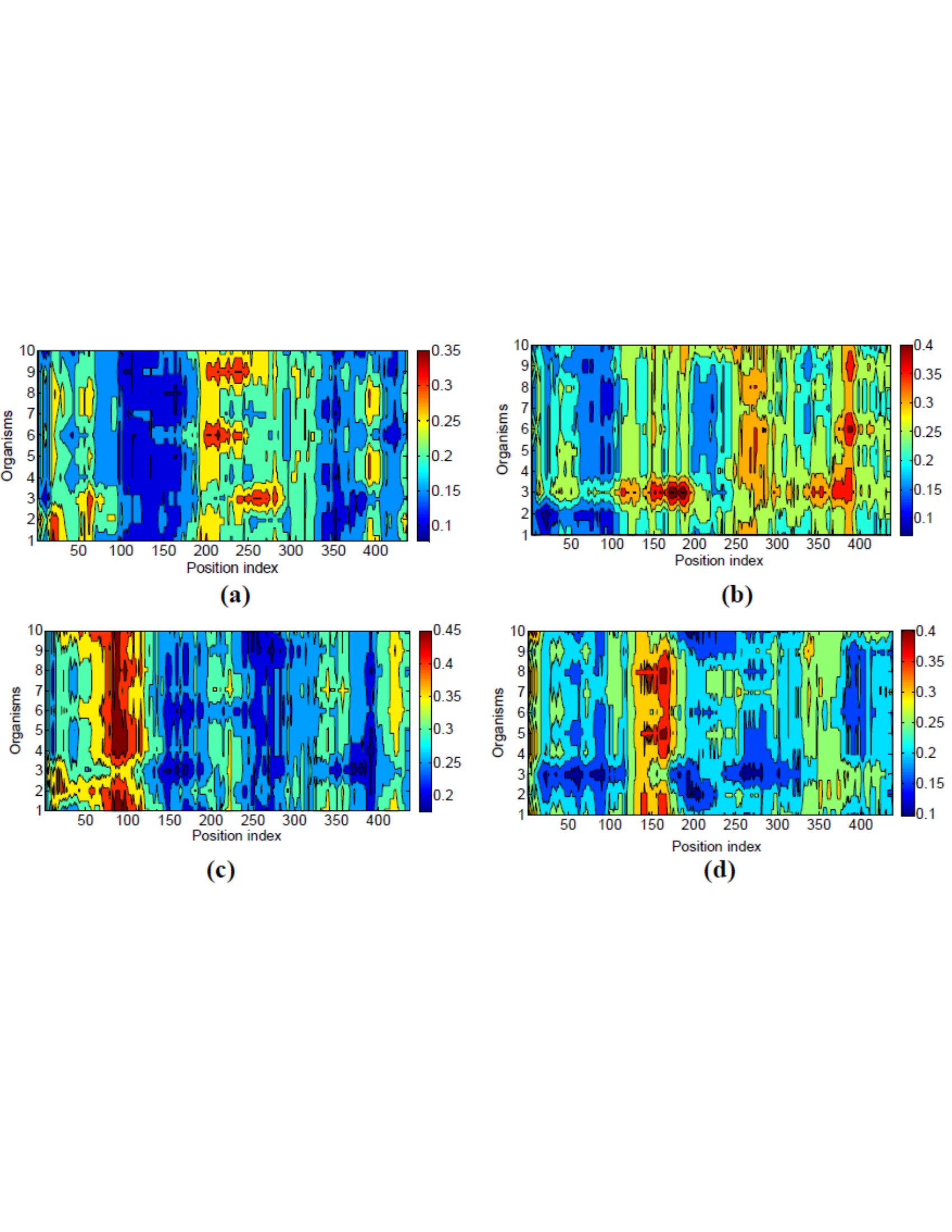

Supplement: Supplementary file 1 — Supplementary Information 1. [file 41598_2021_93154_MOESM1_ESM.tiff]

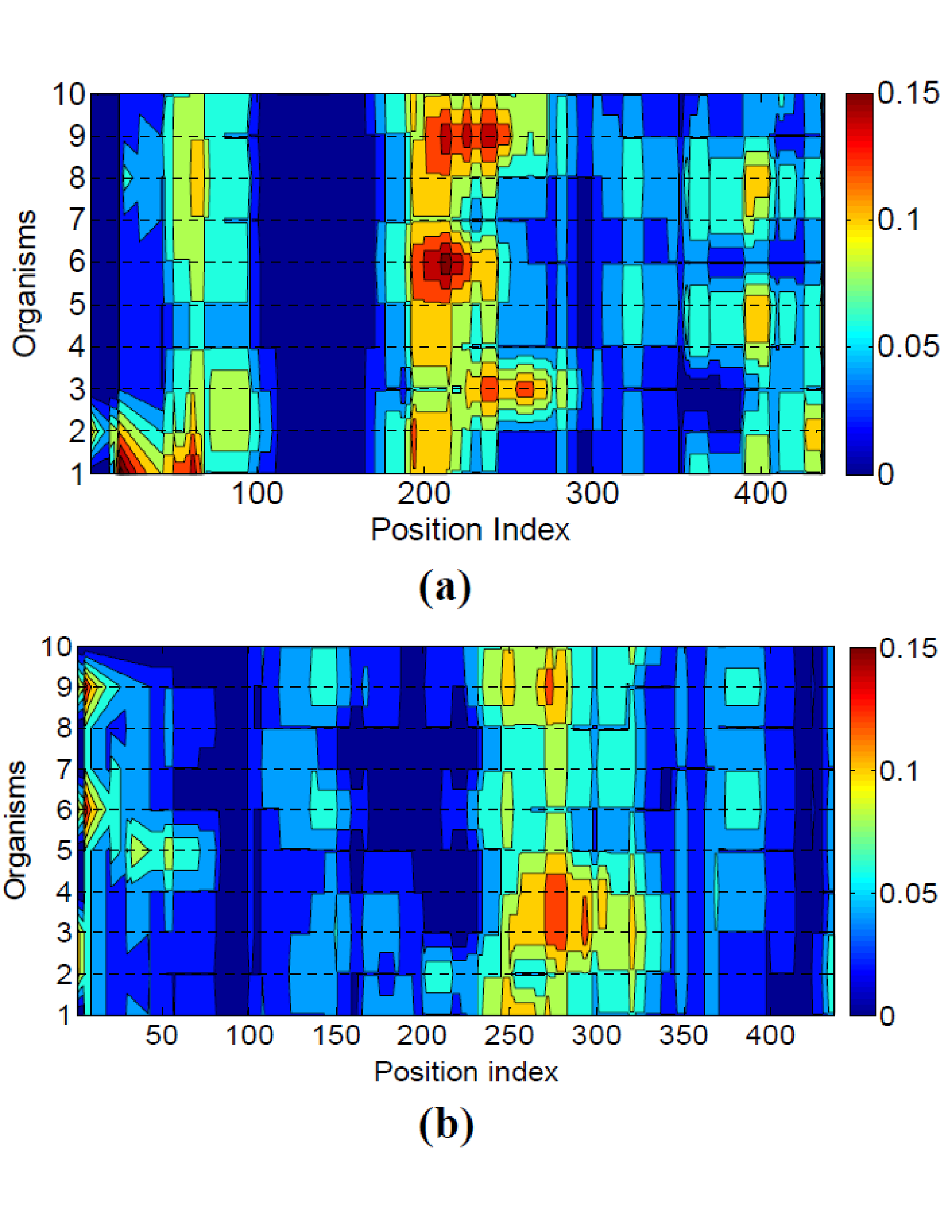

Supplement: Supplementary file 2 — Supplementary Information 2. [file 41598_2021_93154_MOESM2_ESM.tiff]

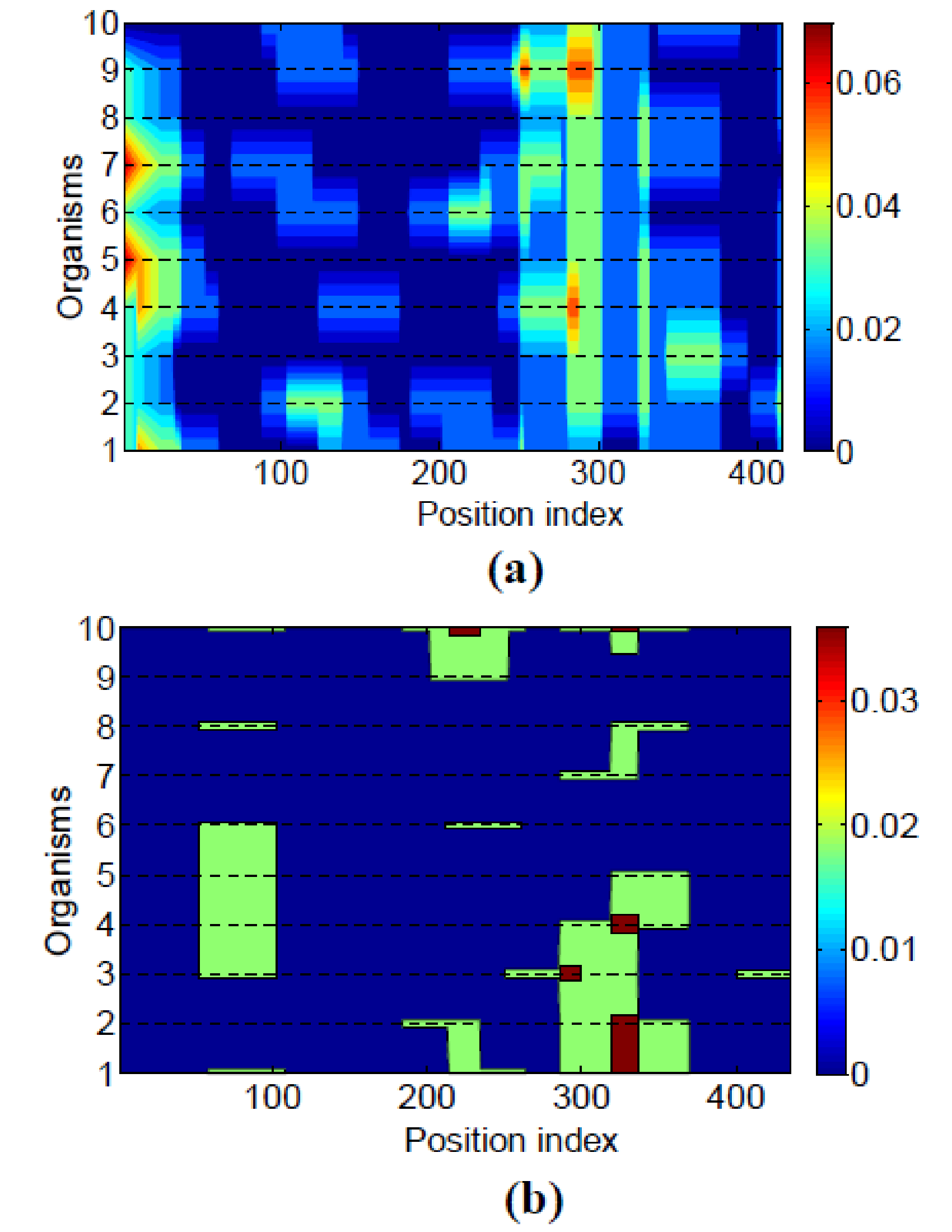

Supplement: Supplementary file 3 — Supplementary Information 3. [file 41598_2021_93154_MOESM3_ESM.tiff]
