# Supplementary material for: Determination of k-mer density in a DNA sequence and subsequent cluster formation algorithm based on the application of electronic filter
Source: Sci Rep. 2021 Jul 1;11:13701. doi: 10.1038/s41598-021-93154-3 (PMC8249421; doi:10.1038/s41598-021-93154-3)
Supplement: Supplementary file 5 — Supplementary Information 5. [file 41598_2021_93154_MOESM5_ESM.docx]

**Supplementary figure:**

**Figure S1.** Mononucleotide density in beta HBB sequences for 10 organisms. From top Nucleotide Density distributions for (a): A, (b): C, (c): G, (d): T is shown. The organisms are 1: *Callithrix jacchus*; 2: *Equus caballus*; 3: *Gallus gallus*; 4: *Homo sapiens*; 5:

*Macaca mulatta*; 6: *Mus musculus*; 7: *Pan troglodytes*; 8: *Papio Anubis*; 9: *Rattus norvegicus*; 10: *Sus scrofa*.

**Figure S2.** Di-nucleotide density in beta HBB sequences for 10 organisms. Nucleotide Density distribution for (a): AA and (b): AC is shown. The organisms are 1: *Callithrix jacchus*; 2: *Equus caballus*; 3: *Gallus gallus*; 4: *Homo sapiens*; 5: *Macaca mulatta*; 6: *Mus musculus*; 7: *Pan troglodytes*; 8: *Papio Anubis*; 9: *Rattus norvegicus*; 10: *Sus scrofa*.

**Figure S3**. Tri-nucleotide density in beta HBB sequences for 10 organisms. Nucleotide Density distribution for (a): ACT and (b): ACG is shown. The organisms are 1: *Callithrix jacchus*; 2: *Equus caballus*; 3: *Gallus gallus*; 4: *Homo sapiens*; 5: *Macaca mulatta*; 6: *Mus musculus*; 7: *Pan troglodytes*; 8: *Papio Anubis*; 9: *Rattus norvegicus*; 10: *Sus scrofa.*

**All the supplementary figure 1 to 3 was drawn by first author B.K.S.**

**Supplementary tables:**

**Table S1**: Algorithm for bin construction.

**Table S2:** Algorithm for bin signature.

**Table S3:** Algorithm for filter operation

**Table S4:** Detail of organisms studied for beta HBB sequences.

**Table S5:** The dissimilarity matrix for beta HBB sequences derived from 10 organisms.

**Table S6:** Detail of organisms studied for HVR-2 sequences.

**Table S7:** Comparison of the existing align based algorithms and our algorithms.

**Supplementary Script S1: Script of Program code.**
