# Supplementary material for: Determination of k-mer density in a DNA sequence and subsequent cluster formation algorithm based on the application of electronic filter
Source: Sci Rep. 2021 Jul 1;11:13701. doi: 10.1038/s41598-021-93154-3 (PMC8249421; doi:10.1038/s41598-021-93154-3)
Supplement: Supplementary file 6 — Supplementary Information 6. [file 41598_2021_93154_MOESM6_ESM.docx]

**Supplementary tables**

Table S1: Algorithm for bin construction

**Algorithm 1** Bin Construction

*B* = {*b* , *b*

, ..... *b q* }

**Input:** q - length of bin **Output:** set of bins *q*

1: 0 ←bincount; 2: 4^q ← n;

3: cell(1,n) ← bin; 4: **for** first = 1:4 **do**



5:

6: **for**qth = 1:4 **do**

1 2 4

7: convert integer to nucleotide character ([first … qth]) ←binq; 8: bincount = bincount + 1;

9: binq← bin{bincount};

10: **end**

11: 

12:

13: **end**

14: bin ←*B_q_*

Table S2: Algorithm for bin signature

**Algorithm 2**Bin Signature

**Input:** Sequence (*seq*), bin (b)**Output:** Bin Signature 1: *m*← length (*seq*);

2: *nbin*← length (*b*); 3:

4:**for***i*←*1….m - (nbin - 1)***do**

5: **if***seq*(*i : i+nbin-1*) = *b* **then**

6: *signature (i) = 1*

7: **else**

8: *signature (i) = 0*

9: **end**

10:

11: *signature* ←*Bin Signature*

Table S3: Algorithm for filter operation

**Algorithm 3**Filter operation **Input:***BinSignature*, *window***Output:** *filter* 1: *w*← length (*window*);

2: *window* = *1/w*array of ones(1,w)*; 3: *0*←*sum*

4: **for***i*←*1….* length (*window*) **do**

5:*make array of zeros with length of i -1* ←*zero*

6: *sum=sum+window (i) * array[zeros BinSignature(1:(length(BinSignature)-(i- 1)))]*

7: **end**

8: *filter* ←*sum*

Table S4: Detail of organisms studied for beta HBB sequences.

| Species | Name | Organism | Accession number | Location |
| --- | --- | --- | --- | --- |
| Primates | Human | *Homo sapiens* | NM_000518.4 | 51- 494 |
|  | New World monkey | *Callithrix jacchus* | XM_002754891.1 | 1 - 444 |
|  | Chimpanzee | *Pan troglodytes* | XM_003312882.1 | 216 - 659 |
|  | Old World monkeys | *Macaca mulatta* | NM_001164428.1 | 10 - 453 |
|  | Baboon | *Papio anubis* | NM_001168847.1 | 1- 444 |
| Ungulates | Horse | *Equus caballus* | NM_001164018.1 | 1- 444 |
|  | Wild boar | *Sus scrofa* | NM_001144841.1 | 53 - 496 |
| Rodents | House mouse | *Mus musculus* | NM_008220.4 | 55 - 498 |
|  | Brown rat | *Rattus norvegicus* | NM_033234.1 | 48 - 491 |
| Birds | Red jungle fowl | *Gallus gallus* | NM_001081704.1 | 1- 444 |

Table S5: The dissimilarity matrix for beta HBB sequences derived from 10 organisms.

| **Organism A B** | **C** | **D** | **E** | **F** | **G** | **H** | **I** | **J** |
| --- | --- | --- | --- | --- | --- | --- | --- | --- |
| **A** 0.000 0.167 | 0.360 | 0.063 | 0.070 | 0.208 | 0.097 | 0.077 | 0.191 | 0.178 |
| **B** 0.000 | 0.340 | 0.151 | 0.159 | 0.228 | 0.159 | 0.164 | 0.225 | 0.154 |
| **C** | 0.000 | 0.328 | 0.328 | 0.380 | 0.366 | 0.324 | 0.393 | 0.340 |
| **D** |  | 0.000 | 0.041 | 0.189 | 0.077 | 0.049 | 0.189 | 0.167 |
| **E** |  |  | 0.000 | 0.181 | 0.080 | 0.011 | 0.175 | 0.164 |
| **F** |  |  |  | 0.000 | 0.211 | 0.183 | 0.082 | 0.219 |
| **G** |  |  |  |  | 0.000 | 0.077 | 0.189 | 0.178 |
| **H** |  |  |  |  |  | 0.000 | 0.178 | 0.162 |
| **I** |  |  |  |  |  |  | 0.000 | 0.205 |
| **J** |  |  |  |  |  |  |  | 0.000 |
| A: *Callithrix jacchus*  B: *Equuscaballus*  C: *Gallus gallus*  D: *Homo sapiens*  E: *Macaca mulatta*  F: *Mus musculus*  G: *Pan troglodytes*  H: *Papio Anubis*  I: *Rattus norvegicus*  J: *Sus scrofa* |  |  |  |  |  |  |  |  |

Table S6: Detail of organisms studied for HVR-2 sequences

Name

|  | | number |  |
| --- | --- | --- | --- |
| *Berber* | Homo sapiens | AY882408.1 | 57- 393 |
| *Chinese* |  | AF346972.1 | 57- 393 |
| *Georgean* |  | AF346982.1 | 57- 393 |
| *Yoruba* |  | AF347014.1 | 57- 393 |
| *Neanderthal* |  | AF142095.1 | 1-337 |
| *Pan_troglodytes_1* | Chimpanzee | JN191222.1 | 16047 - 16383 |
| *Pan_troglodytes_2* |  | JN191223.1 | 16051 - 16387 |
| *Pan_troglodytes_3* |  | JN191224.1 | 16048 - 16384 |
| *Pan_paniscus_1*  *Pan_paniscus_2* | Pygmy chimpanzee | D38116.1  NC_001644.1 | 16046 - 16382  16046 – 16382 |
| *Gorilla_gorilla_1* | Gorilla | D38114.1 | 15942 - 16278 |
| *Gorilla_gorilla_2* |  | NC_001645.1 | 15942 - 16278 |
| *Gorilla_gorilla_gorilla* |  | X93347.1 | 15979 - 16315 |
| *Pongo_pygmaeus_abelii_1*  *Pongo_pygmaeus_abelii_2* | Sumatran orangutan | X97707.1  NC_002083.1 | 16080 - 16416  16080 - 16416 |
| *Pongo_pygmaeus_1* | Borneanorangutan | NC_001646.1 | 15990 -16326 |
| *Pongo_pygmaeus_2* |  | D38115.1 | 15990 -16326 |
| *Hylobates_lar* | Gibbon | NC_002082.1 | 16035 - 16371 |

Organism

Accession

Location

Table S7: Eigenvectors and proportion of variance in PCA

| *Sequence. No. | Eigenvalues of the covariance | | |  | Percentage of the total variance | | |
| --- | --- | --- | --- | --- | --- | --- | --- |
|  | PC1 | PC2 | PC3 |  | PC1 | PC2 | PC3 |
| 1 | 1.897 | 0.725 | 0.378 |  | 63.223 | 24.166 | 11.612 |
| 2 | 1.843 | 1.102 | 0.413 |  | 54.894 | 32.821 | 11.284 |
| 3 | 1.810 | 1.194 | 0.729 |  | 48.495 | 31.973 | 18.533 |
| 4 | 1.901 | 0.677 | 0.424 |  | 63.345 | 22.541 | 13.114 |
| 5 | 1.686 | 0.778 | 0.470 |  | 57.475 | 26.506 | 15.019 |
| 6 | 1.757 | 0.837 | 0.424 |  | 58.216 | 27.728 | 13.056 |
| 7 | 1.834 | 0.727 | 0.308 |  | 63.932 | 25.340 | 9.727 |
| 8 | 1.690 | 0.879 | 0.432 |  | 56.315 | 29.286 | 13.399 |
| 9 | 1.652 | 0.736 | 0.459 |  | 58.031 | 25.851 | 15.118 |
| 10 | 2.146 | 0.740 | 0.678 |  | 60.226 | 20.759 | 18.015 |

*The sequences are 1: *Callithrix jacchus*; 2: *Equus caballus*; 3: *Gallus gallus*; 4: *Homo sapiens*; 5:*Macaca mulatta*; 6: *Mus musculus*; 7: *Pan troglodytes*; 8: *Papio Anubis*; 9: *Rattus norvegicus*; 10: *Sus scrofa*.
